# Innovation in urban agriculture: Evaluation data of a participatory approach (ROIR)

**DOI:** 10.1016/j.dib.2016.04.036

**Published:** 2016-04-21

**Authors:** Felix Zoll, Kathrin Specht, Rosemarie Siebert

**Affiliations:** a Leibniz Centre for Agricultural Landscape Research, Institute of Socio-Economics, Eberswalder Straße 84, 15374 Müncheberg, Germany; b Humboldt-Universität zu Berlin, Germany

**Keywords:** Online survey, Stakeholder, Roadmapping process, Knowledge, Network, Acceptance

## Abstract

The data in this article represent an evaluation of a participatory process called Regional Open Innovation Roadmapping (ROIR). The approach aims at the promotion of regional development. In this case, it was carried out to develop a specific innovation in the field of ‘Zero-acreage farming’ (ZFarming), which is a building-related subtype of urban agriculture. For the evaluation of the process, an online survey was sent to the 58 participants of the ROIR on March 4, 2014. The survey ended on April 8, 2014, and a response rate of 53.54% resulted in a sample size of 31 respondents. The survey was divided into seven different blocks. We analyzed the ROIR process׳s contribution to knowledge generation, the establishment of networks among the participants, the implementation of new projects related to ZFarming, and the increase of acceptance of ZFarming and the selected ZFarming innovation. Furthermore, other remarks, and personal information were collected. Hence, the objective of the survey was to assess whether ROIR is a useful tool to promote the aforementioned innovation drivers, and thereby, the selected innovation, which was developed throughout the process. The data were used in the research article “Application and evaluation of a participatory “open innovation” approach (ROIR): the case of introducing zero-acreage farming in Berlin” (Specht et al., 2016) [1].

**Specifications Table**

**Table:** 

Subject area	*Socio-economics*
More specific subject area	*Technology roadmapping, Open innovation, Urban agriculture, ZFarming*
Type of data	*Excel tables*
How data was acquired	*Through an online survey using* QuestBack EFS Survey 10.3 software
Data format	*Raw and processed data*
Experimental factors	*Ex-post evaluation of a participatory process (ROIR)*
Experimental features	*Data include information on the ROIR׳s influence on knowledge generation, the generation of new networks, the implementation of new projects, the acceptance of ZFarming and the functioning of the ROIR method.*
Data source location	*Berlin, Germany*
Data accessibility	*Data are with this article*

**Value of the data**

● The data are useful to further analyze the impact of ROIR on innovation drivers, such as knowledge generation, the establishment of stakeholder networks, the impact on the acceptance of the innovation and the implementation of new projects.
● The data can be used as a benchmark to compare the evaluations of the implementation and outcomes of future ROIR processes.
● The assessment of the ROIR method can be used to structure and organize future ROIR processes and, hence, improve the conduction of such processes.

## 1 Data

This article contains raw and processed data related to research published by Specht et al. [1]. The data were obtained through an online survey among stakeholders involved in a Regional Open Innovation Roadmapping (ROIR) process [2], [3]. The data is presented in an Excel-file (xls), which contains three data sheets: i) raw survey data (partly coded and supplemented by averages and sums); ii) default variables and iii) codebook, containing the original set of survey questions and answers. The statistics of the evaluation are shown in Table 1 (see also Table 1 in [1] for empirical basis).

## 2 Experimental design, materials and methods

ROIR is a participatory approach to foster sustainable development in a region through developing sustainable innovations [2], [3]. ROIR was applied to map out a plan for the introduction and implementation of an innovative form of building-related agriculture called ‘Zero-acreage farming’ (ZFarming). ZFarming includes open rooftop farms, rooftop greenhouses, vertical farming or indoor farming and typically does not require additional agricultural acreage [4]. It is a relatively new concept, which is why there are still many barriers and research gaps [5]. Consequently, ZFarming is a good candidate for the ROIR approach. The most important aspects that have to be considered when implementing a ZFarming project were elaborated during the ROIR process [3]. The process took place from 2011 to 2013 and was divided into five workshops.

The objective of the evaluation of the ROIR was to assess the process׳s impact on innovation drivers, the general method used, and the future prospects of ZFarming. The ex-post evaluation of the ROIR process was carried out in 2014 through an online survey. The evaluation started on March 4, 2014. Personalized emails with invitations to participate in the survey were sent to all 58 ROIR participants. The emails contained a link that led directly to the survey. In addition, the option to complete the survey on paper was provided. To obtain a high number of completed surveys, two reminders were sent (March 19 and April 1, 2014). The evaluation ended on April 8, 2014.

In accordance with the objective, the survey consisted of 29 questions structured along the following seven topical sections:

I. General perception of ROIR
II. Knowledge generation and exchange
III. Establishing new stakeholder networks and cooperation
IV. Implementation of new projects
V. Acceptance of ZFarming
VI. Other remarks
VII. Personal information

The survey participants were representatives from the following different stakeholder groups: a) activists and projects, b) lobby groups and unions, c) planning and construction, d) policy and administration, e) research and f) sales and distribution, who participated in the ROIR process [3]. These groups were involved because they were identified as the groups most relevant to the practical implementation of ZFarming. The empirical basis consisted of 31 respondents, 19 male and 12 female (see also Table 1 in [1]).

Throughout the survey, different categories of questions were asked, and the participants had to evaluate most statements on a five-point scale ranging from 1=fully agree to 5=fully disagree. If the respondent was not able to rate the statement, the option “I do not know” was given. Additionally, there was a section of multiple choice questions combined with a free text field, which could be used if the given answer choices were not satisfactory. There were also questions that required the participant to select one answer out of several options. In this category of questions, an option to use a text field for answers that differed from the given options was provided as well. To ensure high validity and reliability of the data, the survey was pre-tested by three participants. QuestBack EFS Survey 10.3 (QuestBack GmbH, Cologne, Germany/QuestBack AS, Oslo, Norway) was used to conduct the online survey. The technical functionality of the survey was tested several times by eight people. The raw survey data was exported from QuestBack to Excel (xls) and processed for further analysis.

## Acknowledgment

Funding from the German Federal Ministry of Education and Research (BMBF) supported parts of this study (Funding code FKZ 16I1619). The Leibniz Center for Agricultural Landscape Research (ZALF) is institutionally funded by the Federal Ministry of Food and Agriculture (BMEL) and the Ministry for Science, Research and Culture of the State of Brandenburg (MWFK).

We wish to express our greatest thanks to all the stakeholders in the ZFarm network who collaborated with us throughout the entire process and helped us bring it to fruition.

## Figures and Tables

**Table 1:** Statistics of the survey.

Responses		
	Absolute numbers	Percent
Total sample	58	100.00%
Adjusted total sample	38	65.52%
Completed surveys	31	53.54%
Incomplete surveys	7	12.07%
Time		
Medium completion time (arithmetic mean)	11 min 9.77 s	
Medium completion time (median)	10 min 38.5 s	
